# PC-PNDT - Part I

**DOI:** 10.4103/0971-3026.38500

**Published:** 2008-02

**Authors:** Jignesh Thakker, Bijal Jankharia

**Affiliations:** PNDT Co-ordinator, IRIA, Mumbai, Maharashtra, India; 1 Radiologist, IRIA, Mumbai, Maharashtra, India

The Prenatal Diagnostic Techniques Act of 1994 and its subsequent amendment in 2003 as the Preconception and Prenatal Diagnostic Techniques (prohibition of sex selection) Act (PC-PNDT Act) were brought into force to stop female feticide.

As members of the radiological fraternity and as responsible citizens and members of society it is our duty to not indulge in unethical and unlawful practices. We, as sonologists, have to prove a point here, not only to society but also to the authorities that USG is used not only for diagnostic purposes but also as a life-saver in many cases.

It has been observed that many of our colleagues are not well versed with the PC-PNDT law, which can get them into unnecessary trouble with the authorities. We do not intend to print the whole Act but just to present the basic requirements in a simplified manner. In the first part of this series, we have made a list of DOs and DON'Ts, which will make it easier for radiologists to follow the legal PC-PNDT Act.

## DOs

1. Registration of the clinic is mandatory. Only one registration per clinic is required (even if there is more than one machine). Details of all machines and all radiologists (along with copies of their certificates) should be submitted.
2. In case of portable machines, if the USG is to be done in a vehicle, registration of the vehicle is required.
3. Copy of the registration certificate must be displayed - one in the machine room and another in the waiting room.
4. Signage, in English and in the local language, must be displayed, indicating that fetal sex is not disclosed in the clinic.
5. Ensure that the number of machines and the radiologists attached to the clinic have been mentioned in the registration certificate or on a separate sheet by the appropriate authorities (AA).
6. Form F must be filled in completely and without any delay as soon as the patient's USG is done. The signed consent of the patient as well as the radiologist's signature are a must. Form G is only for invasive procedures.
7. A monthly report should be submitted to the AA regularly, before the 5^th^ of every month. A copy of the same, with the signature of the AA acknowledging receipt, must be preserved.
8. All copies of Form F and monthly reports should be preserved for 2 years. The referral letters from doctors should also be preserved.
9. PC-PNDT Act booklet must be available in the waiting room.
10. The AA must be informed, in writing, about changes in any machine or radiologist. A copy of the same, with the signature of the AA, should be preserved.
11. If a locum radiologist is appointed, the AA must be informed in advance (with the details of the locum radiologist's registration and MMC certificate).
12. Be cooperative if and when the AA visits your clinic to examine the records.

## DON'Ts

1. DO NOT DISCLOSE THE SEX of the child to anyone - under any pressure or in any circumstances.
2. Do not start a USG clinic without PC-PNDT registration.
3. Do not visit any clinic or hospital for USG unless it is registered for PC-PNDT, even if it is for non-obstetric reasons; exception can be made when it is for a very urgent/life-threatening case, but this has to be proved by documentation.
4. Registration certificates are nontransferable. Do not give your certificate to anyone or any place unless you are visiting it regularly.
5. Do not give an experience certificate to anyone.
6. Do not get scared by anyone if you are following all the rules as per the PC-PNDT law.
7. Do not allow anyone to check your records unless the person is accompanied by the AA.

In the second part of this series, in the next issue, we will discuss more details related to Form A and Form F.

